# Action spectrum for reorientations in bacteriorhodopsin of purple membrane in suspension

**DOI:** 10.1038/s41598-023-35121-8

**Published:** 2023-05-16

**Authors:** Hamdy I. A. Mostafa

**Affiliations:** Department of Biophysics, Faculty of Science, Cairo University, Giza, 11757 Egypt

**Keywords:** Biochemistry, Biophysics, Nanoscience and technology

## Abstract

In the present study, the dependency of purple membrane (PM) dielectric responses on the wavelength of light in the range 380–750 nm has showed meaningful changes about the rotation of PM in suspension and about the rotation of bacteriorhodopsin (bR) trimer inside PM, as well. The action spectrum of PM random walk substantiates the existence of two states of bR. One of them (blue edge-state) lies at the blue edge and the other (red edge-state) at the red edge of the visible absorption of bR. The results might bear on correlation of these bands to some bR photocycle intermediates or bR photoproducts. The results implicate the protein–chromophore interactions that eventually underlie protein–lipid interactions. Disrupting the protein–lipid contact during the illumination with light of wavelength in ranges of (410–470 nm) and (610–720 nm) has resulted in emergence of distinct dielectric dispersion at 0.06–0.08 MHz which is comparable to the size of bR trimer or monomer.The work reports on the chromatic adaptation of bR in view of the dielectric spectral parameters of PM. It aimed to explore a correlation seemingly found between the light wavelength and the relaxations of bR trimer inside PM. Changes in rotational diffusion of bR trimer upon blue and red light illumination can influence the three dimensional data storage based on bR, which may implicate bR in bioelectronics.

## Introduction

The photochromic bacteriorhodopsin (bR)^1^ is the sole protein found in the purple membrane (PM) of the archaeon *Halobacterium salinarum* (*Hs*). bR is one of the retinal protein family which has the same topology of G-protein, i.e. consisting of 7 α-helices, that is why bR has potential interest in the field of signal transduction^2^. Moreover, by virtue of possessing photochromic, photoelectric and unique stability properties, bR became attractive in the field of bioelectronics^3^. The present study has showed that bR has two states depending on the wavelength of the illuminating light. The results suggest that lipids have an essential role in appearing of such two states. Lipids have crucial role in the functionality of bR. PM lipids^4,5^ together with bR trimers form a hexagonal structure. It is noteworthy that lipids, in general, are characterized by polymorphism. Alterations in the random walk of PM are related to alterations in the geometry of PM. Such external alterations may reflect concomitant alterations in the internal arrangement of PM. However, studies toward the global structure of PM fragments may clarify previous unresolved behaviours rather than just studying the local fine structure. As a non-optical technique, the dielectric spectroscopy^6^ in its time-independent measurements has enabled one to monitor reorientations in PM and to have an action spectrum of PM chromatic responses.

The photochromic bR acts as a proton pump. The pumping of protons is carried out through photochemical cycle^7,8^ initiated inside bR and comprised of simply five intermediates represented as follows: (where the subscript is referred to the wavelength at maximum absorption) BR_568_ → K_610_ → L_550_ → M_412_ → N_550_ → O_640_ → bR_568_. The vectorial transport of the proton could be detected as an electric signal^9,10^. The action spectrum due to the electric signal in bR was studied^11^ and displayed a band that followed the absorption band of bR. The action spectrum in the present study has been found to substantiate the existence of two steady states of bR. One of them lies at the blue edge (call it as “blue edge”-state) and the other at the red edge (call it as “red edge”-state) of the visible absorption of bR. Therefore, it was an interest in the present paper to report on the wavelength dependence of dielectric responses of PM suspension in its steady state beyond the temporal resolution of the bR photocyle of proton pumping.

## Materials and methods

The bR of PM of *Halobacterium salinarum* was obtained from Sigma Co. A stock solution of PM (of 5 µM) is adjusted, based on molar extinction coefficient^12^ (at 568 nm) of 63,000 cm^−1^ M^−1^. The measurement on PM is carried out in distilled water at ambient temperature and neutral pH. For dark adaptation purposes, the PM suspension stock is divided into groups which are incubated in dark at the ambient conditions. PM suspension should be kept sufficiently in dark (intentionally for three weeks) for dark-adapted sample. However, it is important to determine, for the sample of interest, its own dark incubation prior to measurements.

The sample of bR is connected through a Test Fixture (Hioki 9261) to LCR analyzer (Hioki 3532) interfaced, via RS-232C cross cable, to a personal computer. The level setting, as provided by Hioki 3532, can be open-circuit voltage (V), constant voltage (CV), or constant current (CC). The constant voltage (CV) mode has been selected for both light-adapted and dark-adapted state of purple membrane suspension. The voltage set has been the same for both light- and dark-adapted samples at 1 V.

The dielectric data are recorded in the frequency range from 42 Hz to 5 MHz. The measurement is carried out by simultaneous recording of parallel capacitance (C) and resistance (R) of the sample solution. A cell made of glass whose electrodes made of platinized platinum is used for the dielectric measurements. The value of cell constant (K) could be determined by using methanol solution to be 0.0265 m^−1^ which is kept constant during the process of curve fitting for the measured data of C and R. The setup of measuring electrode is made appropriate so as to minimize the impedance of the electrode polarization^6,13,14^. The residual of electrode polarization impedance is regarded in the iterations carried out to fit the experimental data.

A homemade-setup, which exploits a grating monochromator together with its light system dismantled from conventional visible spectrophotometer, is used to illuminate entirely the PM suspension during the chromatic incubation for a period of time of 5 min. The illumination was carried out with spectrophotometer tungsten lamp, followed immediately by recording C and R. During this recording, the illumination is continued with the same wavelength too. The same steps are done at each wavelength of interest in the range 380–750 nm, provided that the dark-adapted sample is kept in the dark envelope of sample holder away from even dim daylight. Having sensitivity to optical disturbances could stimulate one to be more aware with measuring in dark-adapted states.

## Results:

**Figure 1:**
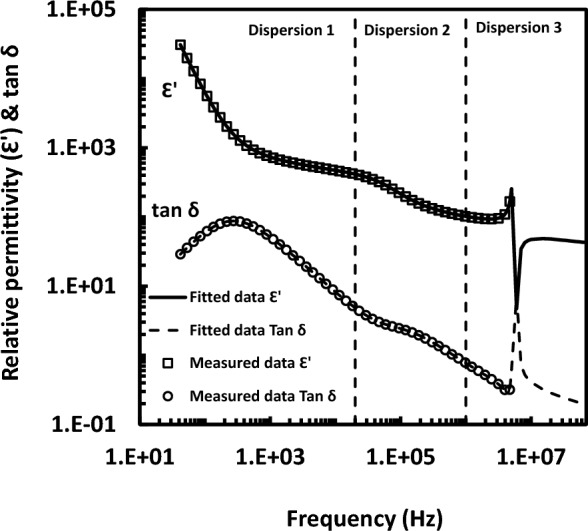
The assignment of dielectric dispersions showing fit line through the experimental data points. Dispersion 1: the rotation of purple membrane in suspension, dispersion 2: the rotation bR trimer or monomer inside purple membrane and dispersion 3: the orientation of retinal or other entity inside bacteriorhodopsin.

In this report, dielectric measurements were presented for bR in its natural membrane (PM), i.e. not reconstituted in membrane. The dielectric approach was used to investigate the rotation of bR in PM, retinal in bR and PM in suspension. It is well known that the PM forms two-dimensional hexagonal crystalline lattice in the plane of the membrane. The lattice consists of bR clustered in trimers. The bR monomer is in shape of a meniscus (i.e. three meniscuses forming one circle per trimer). bR together with lipid molecules form the lattice. Ten lipid molecules were found per one bR monomer^15^. Lipids fill in the spaces found between bR monomers and between bR trimers. As it is known, bR absorbs light maximally at 568 nm in its light-adapted state (B-state) and returns back, spontaneously in dark by thermal isomerization, to its dark-adapted state (D-state) where it absorbs light maximally at 558 nm. The light in both states (B and D) is converted into molecular changes, upon its absorption by bR. These changes are sensed throughout the PM as a consequence to light energy absorption. In other words, the molecular changes could be manifested as changes in the dielectric function of PM. The determinants of the wavelength at maximum absorbance of bR differ in their strengths, underlying alterations in the bR dielectric function. Accordingly, it is reasonable to anticipate that light-induced dielectric response in bR is dependent on the wavelength of the illuminating light. The dielectric response of bR inside PM in suspension has been displayed in Fig. 1. As it is evident from Fig. 1, there are three dispersions labeled as 1, 2 and 3 describing the dielectric function of PM, in the frequency range (42 Hz–5 MHz). Dispersion 1 (at around 1–20 Hz) is assigned to rotation of the fragments of PM in suspension. PM could be curled or flat patches^16^ according to the circumstances that govern the PM state. Dispersion 2 (at around 0.06–0.08 MHz) is assigned to the rotation of the bR trimers or bR monomers inside PM. The protein mobility inside membrane should have biological significance that consistent with the in vivo physiological functions of bR. As to the third dispersion that lies at around 2–20 MHz, it is attributed to dispersion of some group inside PM or to the chromophore (retinal) inside bR. Such assignments in Fig. 1 show the first and second dispersions in normal cases; viz*.* Debye normal absorption dispersion, whereas the third one in an anomalous dispersion; viz*.* resonance absorption case. However, molecules can be considered as elastic systems of electric charges. They can be considered as harmonic oscillators. When the exciting frequency matches with the oscillator natural frequency, the resonance will occur. The characteristic curve that describes the relation between the real part of the dielectric permittivity and the angular frequency (ω) at the resonance is defined in studies of dielectric as an anomalous dispersion (as it is seen from dispersion 3 in Fig. 1).

**Figure 2:**
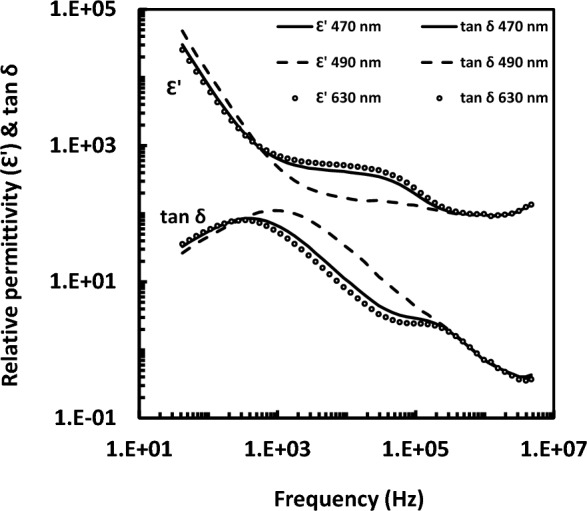
Dielectric spectra measured at blue and red light illuminations. The figure shows the relative permittivity and loss tangent (tan δ) of purple membrane containing bacteriorhodopsin.

The dielectric responses of bR-containing PM in the frequency range 42 Hz–5 MHz in dependence of the wavelength of the illuminating light in the range 380–750 nm have been measured and some of them have been typified in Fig. 2. An observation should be noted in the present study that upon illuminating the D-state with light in the range of (460–470 nm) or light in the range of (630–720 nm), a distinct dielectric dispersion (dispersion 2) is emerged around 0.06–0.08 MHz. This distinct dispersion was found at illumination with other wavelengths, as well, but with very low dielectric strengths (e.g. at 490 nm) as it is seen in Fig. 2. The measured data points of R and C are fitted accordingly to one or two normal dispersions (with Cole model^17^) plus an additional term for the resonance anomalous dispersion. The curve fitting of both R and C was run simultaneously. As it is shown in Fig. 1, fitted lines go through the measured data points of both the real part of the dielectric permittivity and the dissipation factor (or loss tangent, tan δ). It should be noted here that the fitted line is extended beyond the measured data points in case of the last dispersion. From the curve fitting process, one could get many dielectric parameters belonging to the three dispersions. Only the resonant frequency of the dispersions has been chosen to represent the action spectrum.

**Figure 3:**
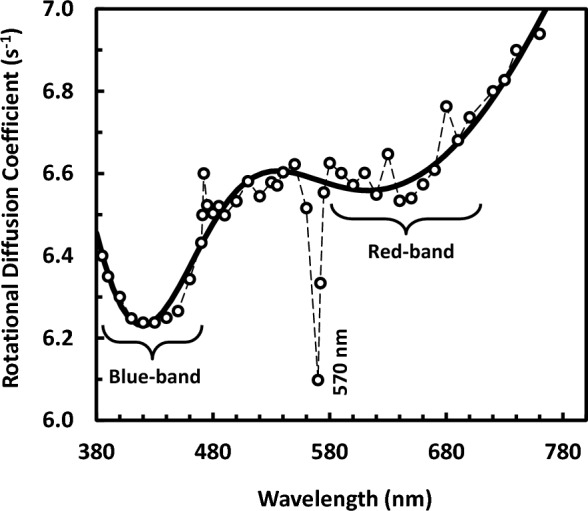
Action spectrum due to the rotational diffusion coefficient (D_r_), in s^−1^, of purple membrane in suspension. The thick solid line going through the data points is due to Gaussian fitting, whereas the thin dashed line is just to guide the eye for spikes superimposed on the curve which have been excluded from the curve fitting procedure. According to the Gaussian fitting, the blue-edge and red-edge bands are at 410 nm and 620 nm, respectively.

As it is known, the characteristic frequency of an orientational dispersion determines the rotational diffusion coefficient of the rotated entity. An estimated figure for PM rotational diffusion coefficient (D_r_) could be obtained. If one takes in consideration that the dichroism (or birefringence) decays with relaxation time equal to one-third that of the dielectric dispersion (τ), the rotational diffusion coefficient can be written as D_r_ = 1/(2τ). Therefore, from the resonant frequency of dispersion 1, one could estimate the value of the rotational diffusion coefficient for PM in suspension, as it is seen in Fig. 3. The data in Fig. 3 are fitted well into Gaussian distribution with two bands (blue and red bands); the data of spikes superimposed on the curve were excluded from the fitting procedure. According to the Gaussian fitting, the blue-edge and red-edge bands are at 410 nm and 620 nm, respectively. As to dispersion 2, the rotational diffusion coefficient of bR trimer in PM could be determined. This rotation of trimer is most likely around the normal to PM^18^. The rotational diffusion coefficient of bR about normal to membrane was already determined to be of 0.23 × 10^4^ s^−1^ at 22 °C for reconstituted apo-brown membrane of Halobacterium *halobium*^19^, while it was 0.23 × 10^4^ s^−1^ and 1.1 × 10^5^ s^−1^ for protein: lipid ratio of 1.69 and 0.25, respectively, at 25 °C for bR reconstituted in lipid vesicle with different protein : lipid ratio^20^. In other work^21^, it was found that the values of rotational diffusion coefficient of bR reconstituted in lipid system fall within the range of 5–10 × 10^4^ s^−1^. These values of rotational diffusion coefficient are in comparison with an average value of 60 × 10^4^ s^−1^ determined in the present study. It should be noted that the present value is for bR rotation in native lipids of PM; not in reconstituted membranes. Obviously, the present result is larger one order of magnitude. The rotational diffusion coefficient depends on the square of size of molecule in the plane of membrane. In addition, the viscosity of lipid in PM could account for the present results. The membrane viscosity depends strongly on the protein concentration. The aggregational states of bR in reconstituted membrane system and in PM are quite different. The absence of retinal in apo-brown membrane reconstitution might account for the present result, as well.

**Figure 4:**
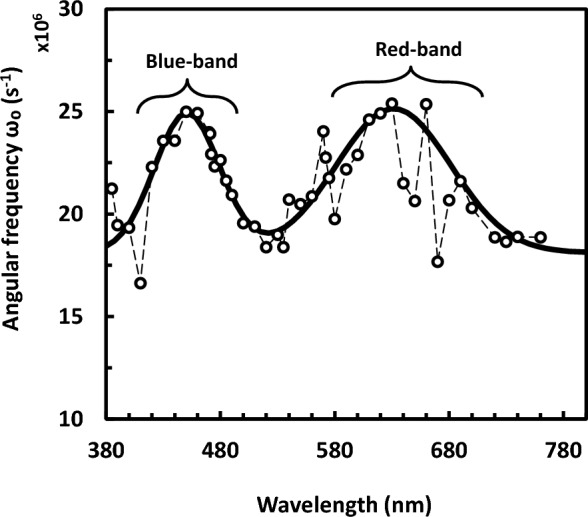
Action spectrum due to the dispersion of retinal or other entity in bacteriorhodopsin. This is represented by the characteristic angular frequency of the dispersion in s^−1^. The thick solid line is due to Gaussian fitting, whereas the thin dashed line is guide to the eye for the little spikes superimposed on the curve which have been excluded similarly from the curve fitting procedure. According to the Gaussian fitting here, the blue-edge and red-edge bands are at 450 nm and 630 nm, respectively.

The wavelength dependence of the resonant angular frequency (ω_o_) belongs to the dielectric dispersion 3, which is assigned to some group or retinal inside bR, is shown in Fig. 4. Similarly, the data in Fig. 4 were fitted into Gaussian distribution like in Fig. 3. According to the Gaussian fitting here, the blue-edge and red-edge bands are at 450 nm and 630 nm, respectively. Accordingly, an average value could be assigned as 430 nm and 625 nm to the position of blue-edge and red-edge bands, respectively. It should be emphasized that the orientation of retinal cannot be determined by dielectric spectroscopy. Figure 4 gives, at least qualitatively, an indirect idea about changes in the retinal orientation. It is reasonable that either conformational changes in bR or rotations of bR are more likely to accompany the retinal reorientation. It should be noted that the reorientation of retinal is restricted, for example, because of the tryptophan residues. One should consider that the existence of bulky tryptophan residues results in possible tight packing of amino acid side chains around both of the polyene chain and the ring of retinal. However, it was noticed that there is an upward movement of the retinal manifested as an increase of 2.2° in the angle of retinal transition moment together with the movement of helix (G) seen by x-ray diffraction of PM^22^. These movements were considered as a compensation of the protein moiety, which are essential as consequences to the photo-isomerization process of the retinal upon absorbing light photons.

The action spectrum (as shown in Fig. 3 and in Fig. 4) does not follow the absorption spectrum of bR. However they do reflect changes-like difference spectrum between two specific bR states. In contrast, the action spectrum of electric signal of bR in PM was found to follow in general its absorption spectrum in a study dealt with measuring the protein electric response signal^11^. However, the protein electric response signal reflects proton pumping functional activity of light adapted bR, while the present experiment is for measurements done in the steady state of bR. The presented action spectrum (as in Figs. 3 and 4) shows instead two continuous bands, one at the blue edge (around 430 nm) and the other at the red edge (around 625 nm) of the visible absorption band of bR, enclosing spike lying at the yellowish green region (around 570 nm) as it is evident from Fig. 3 only. Such Fig. 3 shows action spectrum that is due to the rotation of PM in suspension, whereas Fig. 4 shows action spectrum that might reflect the dispersion of retinal in bR (or other group in PM). Seemingly, these action spectra bear some correlation on the conformational changes occurred during the bR-photoreaction in the monochromatic light-adapted or dark-adapted states.

## Discussion

The retinal as a central unit is an important moiety in the light sensitivity of bR. It is linked inside bR to Lys216 via protonated Schiff base. The protein part (around both of retinal and its active center) is responsible determinant of the wavelength at maximum absorption. This interaction between protein and chromophore underlies the essence of lipid-protein contact (i.e. underlies the essence of existence of bR crystal lattice in PM). It was showed that the bR crystal lattice is important for in vivo physiology of bR^23^. The existence of two states of bR (blue and red-edge states) might be correlated to the bR lattice. The relation between the aggregational state of bR and its activity should be of interest, particularly in the view of the crystal lattice. The aggregational state of bR is dependent on the lipids in PM. Unwinding of lipid-protein contact most probably leads to rotation of bR trimers in PM (or bR monomers in the trimers). The reorientations in PM have been investigated in the present dielectric study for the steady state of bR, beyond the fine details of its photocycle. The trimers reorientations have been observed in the frequency range of (0.06–0.08 MHz). Such range of frequency is comparable to the size of trimer or monomer. It is noteworthy that reorientations in PM were already investigated, but during the photocycle of bR, employing linear dichroism spectroscopy^24–26^ and discussed in terms of trimeric bR lattice, as well.

An observation in the present study is that rotations in PM alter at blue and red wavelengths of the illuminating light. This implies indirect link of the lipid-protein interactions to the determinants of wavelength of light absorption. The underlying of this indirect link is the protein composition itself found within the retinal pocket and around the active center of the chromophore. That is to say, the determinant of the wavelength of absorption, isomerization and bond rotation of retinal can be ensued from the composition of protein. The lipids are in contact with protein; lipids act as glue connecting monomers to each other’s, and also the trimers to each other’s forming by this way two-dimensional hexagonal lattice. Accordingly, lipid would be considered as indirect determinant of the isomerization of retinal based on its initial configuration; viz*. all-trans*, 13 *cis*, or any other conformations. This is on one hand. On the other hand, the retinal has specifications that accommodate well its pocket in the protein matrix. As it is known from literatures, the bR protein catalyzes the isomerization of retinal and it might therefore be stereo-specific. As crucial determinant of bR structure, lipid can be thought to take also part in this protein-catalyzed stereo-specificity. Conceptually, if one looks at protein as an “operating” unit and at retinal as “central” unit, then a “mediating” unit would be introduced to join these two units. This “mediating” unit would be assigned to lipid exclusively by virtue of its paradox behavior, i.e. whilst lipid itself signifies an importance for the existence of bR trimers in lattice; it is also responsible for disassembling of the bR lattice. Inasmuch the bR monomer pumps proton^27,28^, then lipid is supposed to have specific mediating actions in bR lattice in order to render such highly organized PM in a thermodynamically favorable state. Seemingly, PM lipids bring about non-local link with retinal, or more strictly with the pocket of retinal, i.e. its “envelope” within the protein matrix, where the configuration of pocket might differ dependently on the configuration of retinal. It was observed that removal of the chromophore alters the lipid-protein interactions in lipid-vesicle study^29^ and resulted in alterations in the crystalline lattice^30,31^. Even illumination with continuous light caused photobleaching^32^ of bR (i.e. removal of retinal where no hydrolysis reagents were used). These two observations throw light on retinal-lipid non-local link. These views favor the lipid-mediating concept, for instance, in switching between two bR states, e.g. bR lattice assembled/disassembled states. Accordingly, correlation seems to exist between the bR crystal lattice disassembling and the two continuous bands at red and blue light. This can be rationalized in what follows. No solubilizing agent has been used, but it is the action of blue and red light illumination that has resulted in reorientations of BR trimers (or monomers) during the period of bR incubation under light exposure. Different wavelengths of light might result in different accommodations of the retinal pocket in the respect of retinal-protein interactions. As a consequence, the retinal-protein interactions might underlie indirect retinal-lipid interactions, as well. Concomitant unwinding of lipids could lead to reversible disassembling state of bR trimers.

The 75% protein and 25% lipid content (by weight) in the PM might be correlated to the rotational diffusion coefficient shown in Fig. 3, in the sense that the content of lipid together with the configuration state of lipid could be contributed to determine the rigidity of purple membrane. Based on the rigidity of purple membrane, its shape and geometry could be determined in the view of lipid polymorphism. In principle, the shape and geometry are key determinants for the rotational diffusion coefficient. The light-induced changes in PM in terms of lipid-protein and retinal-protein interactions might result in variations in rotational diffusion. These variations are little, but become pronounced, as shown in Fig. 3, upon illumination with blue and red light; most likely as a consequence of the rotation of bR trimers at blue and red light.

As mentioned above, the action spectrum does not follow the absorption spectrum of B-state but it might follow the absorption spectrum of two photoproducts of the photocycle. These two photoproducts would be relevant to the rotation of bR trimer and/or bR monomer inside PM. These photoproducts seem to be relevant to: (a) M-state of bR which absorbs light at 412 nm respective to the present blue edge state and (b) O-state of bR which absorbs light at 640 nm respective to the present red edge state. As it is known, M and O states are intermediates of the photocycle of the light-adapated state of bR. Alternatively, one could consider that these two photoproducts (corresponding to blue and red-edge states) might be related to other blue light-absorbing and red light-absorbing photoproducts, respectively, which may result from photocycle elicited of dark-adapted state of bR. The present study has dealt initially with a dark-adapted sample of bR, which may initiate the photocycle of dark-adapted bR^33^ upon light illumination. Lastly, the above interpretation does not exclude the possibility of neither the so-called blue light syndrome effect^34,35^ nor the red light effect. However, the red light was implicated for causing dark-adaptation in bR^36^; this red light^37^ may be of syndrome effect, too. More study will obviously be needed to elucidate the fine details about these two states of bR; the so-called blue-edge and red-edge states.

## Data Availability

All data are available in the article.
